# A Blind-Identification Test on *Criconema annuliferum* (de Man, 1921) Micoletzky, 1925 Species Complex Corroborate the Hyper-Cryptic Species Diversity Using Integrative Taxonomy

**DOI:** 10.3390/plants12051044

**Published:** 2023-02-24

**Authors:** Antonio Archidona-Yuste, Juan Emilio Palomares-Rius, Ilenia Clavero-Camacho, Carolina Cantalapiedra-Navarrete, Gracia Liébanas, Pablo Castillo

**Affiliations:** 1 Institute for Sustainable Agriculture (IAS), Spanish National Research Council (CSIC), Avenida Menéndez Pidal s/n, Campus de Excelencia Internacional Agroalimentario, ceiA3, 14004 Córdoba, Spain; 2 Department of Animal Biology, Plant Biology and Ecology, University of Jaén, Campus ‘Las Lagunillas’ s/n, Edificio B3, 23071 Jaén, Spain

**Keywords:** cytochrome c oxidase subunit 1 (COI), D2-D3 expansion domains of the large ribosomal subunit (28S), haplotype, internal transcribed spacer (ITS), multivariate morphometric analysis, species separation

## Abstract

Ring nematodes are obligate ectoparasites on crops and natural herbaceous and woody plants, and some species are of economic importance and cause damage to roots of several crops. Recent integrative taxonomical analyses recognized the existence of two cryptic species within the *Criconema annuliferum* morphotype in Spain. In this study, we corroborated that morphometric, morphological and a multi-locus analysis (including the ribosomal markers D2-D3 expansion segments of 28S rRNA, ITS rRNA, 18S RNA, and the mitochondrial DNA cytochrome oxidase I gene) identified a new lineage clearly separated from *C. annuliferum*, *C. paraannuliferum* and *C. plesioannuliferum*. The new lineage was described herein as *Criconema pseudoannuliferum* sp. nov., confirming that *C. annuliferum* species complex species complex comprises a hyper-cryptic species complex. This research analysed soil samples from the rhizosphere of maritime pine (*Pinus pinaster* Ait.) forests in Bermeja-Crestellina Mountain, located at the western part of Málaga province, southern Spain. The integrative taxonomical analyses revealed the occurrence of a new cryptic species identified using females, males and juveniles with detailed morphology, morphometry and molecular markers, described herein as *Criconema pseudoannuliferum* sp. nov. All molecular markers (D2-D3, ITS, 18S and COI) were obtained from the same individual that was also used for morphological and morphometric analyses. This research demonstrated the hidden diversity within the *C. annuliferum* species complex species complex can reach to four lineages under ribosomal and mitochondrial gene markers for one morphospecies group, which includes four species, viz. *C. annuliferum*, *C. paraannuliferum*, *C. plesioannuliferum*, and *C. pseudoannuliferum* sp. nov. *Criconema pseudoannuliferum* sp. nov. was detected in moderate soil density in two maritime pine forests (5 and 25 nematodes/500 cm^3^ of soil) suggesting that does not cause damage to maritime pine.

## 1. Introduction

Ring nematodes of the genus *Criconema* (Hofmänner & Menzel, 1914 [1]) comprise the largest genus within the Criconematidae family, with more than 100 nominal species characterized mainly by smooth body annuli but without appendages in females and sturdy stylet [2]. Several species of ring nematodes are of economic importance and cause damage to roots of several crops [3,4]. A clear example is the significant damage that can be observed in *Prunus* when nematode population densities are high (i.e., 50 specimens/g soil in peach plantations) [5,6]. Moreover, *Criconema xenoplax* is considered as being one of the main factors in peach tree short life (PTSL), a syndrome associated with this nematode, cold injury and *Pseudomonas syringae* pv. Syringae, which results in premature tree mortality [7]. Therefore, an accurate diagnosis is necessary where molecular taxonomy is providing a powerful tool given the great phenotypic plasticity that characterizes criconematid species [8,9,10,11,12,13].

One of the widespread distributed species within *Criconema* comprise *Criconema annuliferum*, as reported in several European countries (*viz*. Austria, Belgium, Bulgaria Netherlands, Poland, Russia, Slovak Republic, Spain) [11,14,15,16,17,18,19], but also in Africa [20], Asia [2,21], New Zealand [22] and South America [23]. Recently, Clavero-Camacho et al. [13], using morphometric and integrative taxonomical analyses, documented the existence of a species complex within the morphotype *C. annuliferum*, including two new species (*C. paraannuliferum* and *C. plesioannuliferum*) from cultivated (mainly *Prunus* species fruit-trees) and a natural environment (common yew, *Taxus baccata* L.), respectively. In Spain, *C. annuliferum* species complex species complex has been recognized in numerous localities including many cultivated (*viz*. *Prunus* plantations, almond, olive, peach, pepper) and few natural environments and forests [13,24,25]. In some cases, *C. paraannuliferum* in *Prunus* plantations reached population densities ranging from 7950 to 42,491 nematodes/500 cm^3^ of soil, which could be pathogenic to plants [13]. As highlighted by Clavero-Camacho et al. [13], the number of cryptic species diversity within *C. annuliferum* species complex species complex is expected to increase in the next few years. mainly due to the use of integrative taxonomy approach for species identification. In view of this, a new extensive survey was carried out in natural environments in southern Spain to study the potential occurrence of new cryptic species complexes within the genus *Criconema*. We detected an unidentified population of *Criconema* which appeared indistinguishable from the detailed morphology of *C. paraannuliferum* and *C. pseudoannuliferum*. This prompted us to perform a blind-identification test within the same nematological team who defined the *C. annuliferum* species complex species complex, and described two new species: *C. paraannuliferum* and *C. pseudoannuliferum* [13].

Recent studies on the molecular variability within *C. annuliferum* species complex have demonstrated very low intraspecific variation on ribosomal genes (D2-D3, ITS and 18S), and low to moderate variation in mitochondrial DNA (COI), including individual species from different geographic areas and environmental conditions [11,13], which may be related to the limited dispersal capacity of these nematodes [2]. This suggests that the variation detected among species of this complex may be the consequence of the phenotypic plasticity of these nematodes [8,9,10,11,12,13].

In recent years, integrative taxonomy using ribosomal and mitochondrial molecular markers revealed that the current distribution of *Criconema* spp. is indeed dominated by genetically divergent and morphologically similar cryptic species complexes, in which diversification is not accompanied by apparent ecological or morphological separation on traditional quantitative traits [13,26,27,28]. This study focused on disentangling and explored if *C. annuliferum* species complex can be composed of a “hyper-cryptic” species complex. “Hyper-cryptic” species complex can be defined herein as morphospecies taxon, which dwells of “several” numbers of valid but undiagnosed species (*viz*., one “species” becomes four or more valid species) [29]. Hyper-cryptic species complexes are likely to be common in several organismal groups, including fish (*viz*. genus *Galaxias*, [29]), crustaceans (genus *Gammarus*, [30]), annelids (*viz*. genus *Terebellides*, [31]) or plant-parasitic nematodes of the genera *Xiphinema*, *Longidorus* and *Paratylenchus* [32,33,34].

The aims of this study were to (i) accurately identify with morphological and morphometrical approaches the new population of *Criconema* detected in forests of maritime pine (*Pinus pinaster* Ait.) in a mountainous area at Málaga province, southern Spain; (ii) disentangle the diversity of *C. annuliferum* species complex through a blind-identification test and consequent integrative taxonomy combining morphological analysis and a species delineation approach based on multivariate morphometric and genetic methods; (iii) describe the new species of the genus *Criconema* belonging to the *C. annuliferum* species complex group; (iv) employ molecular characterization of the sample *Criconema* sp. population using a multi-locus approach including ribosomal (D2-D3 expansion segments of 28S rRNA, Internal Transcribed Spacer region (ITS) rRNA, 18S rRNA) and the mitochondrial cytochrome c oxidase subunit I (COI); and (v) study phylogenetic relationships within Criconematidae using the obtained molecular markers.

## 2. Results

From the ten soil samples of the rhizosphere of maritime pine analysed in the municipality of Casares, Málaga province, southern Spain, ring nematodes of the genus *Criconema* were detected in two samples (20.0%), with population densities ranging between five and twenty-five nematodes/500 cm^3^ of soil, respectively, and all of them were identified under integrative taxonomy as a new cryptic species herein described as *Criconema pseudoannuliferum* sp. nov.

### 2.1. Blind-Identification Test

The results of the blind-identification test, without knowing a priori the results of molecular analyses, are provided herein. After conducting the measurement and morphological identification of the fixed mounted specimens, IAS-CSIC laboratory identified this *Criconema* population as belonging to the recently described species *C. plesioannuliferum*, being coincident or within the range of the main diagnostic traits, as well as the morphology of tail annuli being found to be mostly not bifurcated [13]. The only detected difference in spicules length (47–48 µm vs. 29–32 µm) was considered as an intraspecific variation. The IFAPA laboratory carried out the morphometric analyses of the new *Criconema* population, including all the available *C. annuliferum* species complex populations (see below), concluding that, focusing in morphometry, there could be detected mixed nematode specimens, in this population, from the recently described species *C. plesioannuliferum* and *C. paraannuliferum* [13]. Finally, the UJA laboratory, on the basis of morphology and measurements, as well as SEM observations carried out in this laboratory, identified this *Criconema* population as belonging to *C. plesioannuliferum* [13], detecting only minor differences in spicules length, which was attributable to intraspecific variation. In summary, the threefold blind-identification test based on morphology and morphometry demonstrated that without applying any molecular marker, the new *Criconema* population cannot be separated from other species within *C. annuliferum* species complex.

### 2.2. Species Delimitation Using Morphometry by Principal Component Analysis

**Figure 1:**
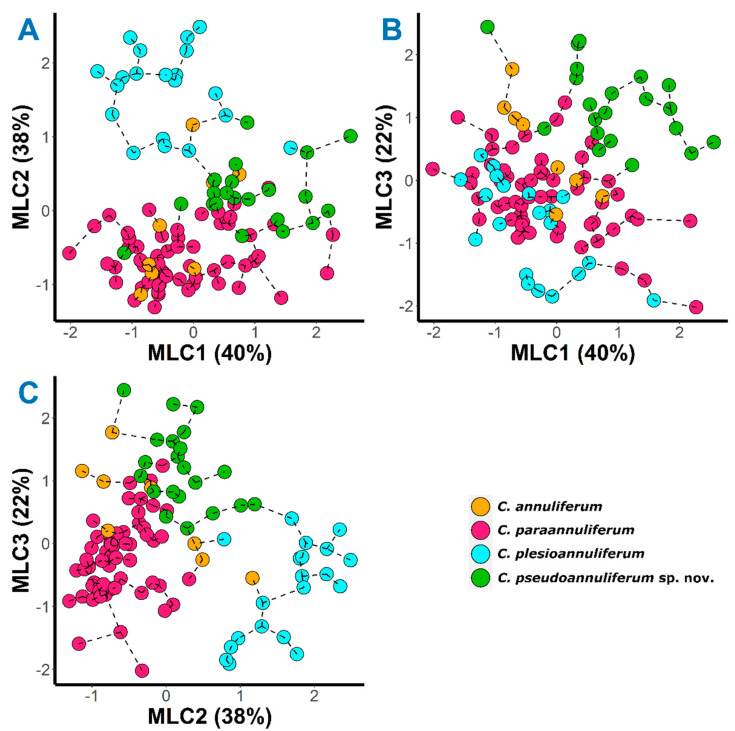
Factor analysis with a superimposed minimum spanning tree on *Criconema annuliferum* species complex. (**A**), maximum likelihood component 1 (MLC1) vs. maximum likelihood component 2 (MLC2); (**B**), maximum likelihood component 1 (MLC1) vs. maximum likelihood component 3 (MLC3); (**C**), maximum likelihood component 2 (MLC2) vs. maximum likelihood component 3 (MLC3).

In factor analysis (FA), we accounted for 70% of the total variance from the morphometric variability of the *C. annuliferum* species complex for the first selected three components (Table 1). Here, we found that the maximum likelihood component 1 (MLC1) was mainly dominated by the nematode body (L) and stylet length, and for the a ratio with high positive correlations (eigenvalues = 0.86, 0.66 and 0.67, respectively). MLC2 was mainly dominated by a number of annuli between the posterior end of body and the vulva (RV), number of annuli on tail (Ran) and for the c′ ratio with high positive correlations (eigenvalues = 0.85, 0.90 and 0.81, respectively). Finally, MLC3 was mainly dominated by a high positive correlation for the V ratio (eigenvalue = 0.64). In short, we detected that the selected three components were related with the overall shape and length of nematode body length, particularly with the posterior part of the nematode as reported by Clavero-Camacho et al. [13]. On the basis of the graphical representation (Figure 1), we noted here that the graphical representation of the *C. annuliferum*-complex closely matched the spatial pattern reported by Clavero-Camacho et al. [13]. We observed that the specimens of all species were projected showing an expanded distribution along the plane for all the projected combinations of the components owing to their wide morphometric variation within species and/or populations, this being more pronounced for *C. paraannuliferum* and *C. annuliferum* as previously shown by Clavero-Camacho et al. [13]. As a consequence, we did not detect a clear separation among specimens belonging to the new *Criconema* population (i.e., *C. pseudoannuliferum* sp. nov. in Figure 1), with those belonging to the species considered within *C. annuliferum*-complex [13]. Although some specimens belonging to the new population remained rather spatially separated when the plane composed by MLC1 and MLC3 was projected (Figure 1), there were several specimens clearly grouped with some belonging to *C. paraannuliferum* and *C. annuliferum*. Therefore, no reliable morphometric evidence was observed to differentiate the new population from those previously described within the *C. annuliferum* complex, particularly for those belonging to *C. paraannuliferum* and *C. annuliferum*. However, and in line with Clavero-Camacho et al. [13], we observed that the most of specimens belonging to *C. plesioannuliferum* were spatially separated from the rest of species within the *C. annuliferum* complex with the exception of the projection on the plane of MLC1 and MLC3 where specimens of all species were randomly plotted (Figure 1). The same pattern described for the factor analysis was observed when we superimposed a minimum spanning tree (MST) on the plots corresponding to the studied combinations of the first three components of the ordination analysis (Figure 1). In short, the integration of the new *Criconema* population in the already described and established species delimitation approach [13] has not led to new insights for the deciphering of species within *C. annuliferum* species complex but rather has further increased the level of complexity within this cryptic species complex (i.e., hyper-cryptic species complex).

### 2.3. Species Separation Based on Ribosomal and Mitochondrial DNA

Species separation using molecular markers demonstrated that *C. pseudoannuliferum* sp. nov. was clearly separated from *C. annuliferum* from Belgium, and *C. paraannuliferum* and *C. plesioannuliferum* from Spain. The intra- and inter-species molecular variation for D2-D3 of all four species was higher than 0.10, except for *C. plesioannuliferum* and *C. pseudoannuliferum* sp. nov., for which it was 0.09 and 0.05, respectively (Table 2), suggesting that the probability of species identification with this gene was low. However, for all four species, ITS and COI genes showed intra- and inter-species molecular variation clearly below 0.10 (Table 2), suggesting that the probability of species separation with these loci was high [36]. Likewise, the P ID (Liberal) values for all four species and locus were ≥0.95, suggesting that species can be adequately separated [37,38]. Additionally, all clade supports for the three loci were well-supported (PP = 1.00), and the Rosenberg’s P_AB_ values also support the monophyly of each species separately [39].

### 2.4. Ribosomal and Mitochondrial Haplotype Diversity within Criconema annuliferum Species Complex

**Figure 2:**
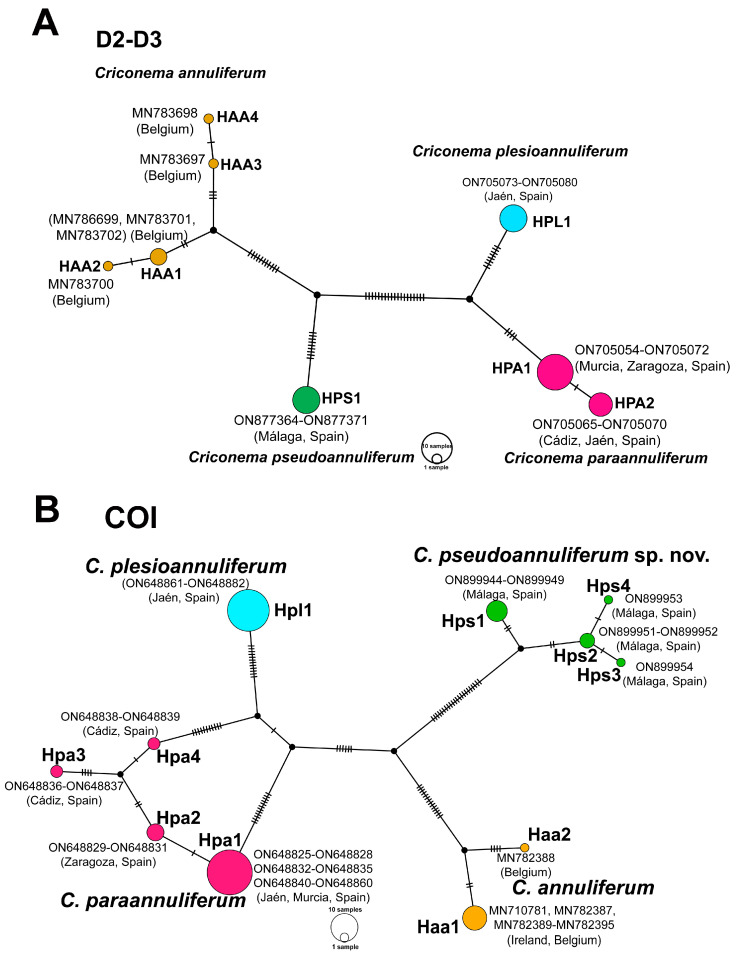
TCS network analysis of D2-D3 expansion segments of the 28S rRNA (**A**), and partial mitochondrial COI (**B**) haplotypes of *Criconema annuliferum* species complex. Coloured circles embody haplotypes and their diameter is proportionate to the number of individuals sharing the same haplotype. Black short lines on the branches specify the numbers of mutate nucleotide in the alignment that separate each haplotype. Small black circles represent missing haplotypes.

The ribosomal and mitochondrial haplotype diversity study in *C. annuliferum* species complex was based on D2-D3 expansion segments of the 28S rRNA and COI, comprising 42 and 80 sequences, respectively (eight and eleven sequences from *C. pseudoannuliferum* sp. nov., twenty and thirty-six sequences from *C. paraannuliferum*, eight and twenty-four sequences from *C. plesioannuliferum*, and six and nine sequences from *C. annuliferum*, respectively). The haplotype network analysis for D2-D3 and COI sequences showed 1 and 4, 2 and 4, 1 and 1, and 4 and 2 haplotypes, respectively, in the *C. annuliferum* species complex (Figure 2). In *C. pseudoannuliferum* sp. nov., a single haplotype (HPS1) was detected in D2-D3, whereas the haplotype Hps1 was most common in COI with seven sequences, Hps2 with two sequences, and haplotypes Hps3 and Hps4 were found only once (Figure 2); all of them were from the rhizosphere of maritime pine (*Pinus pinaster* Ait.) at Málaga province (Spain). In *C. paraannuliferum*, two haplotypes were detected in D2-D3 (HPA1 and HPA2) with fourteen and six sequences, respectively, from the rhizosphere of *Prunus* plantations in Murcia and Zaragoza provinces (Spain), and from the rhizosphere of wild olive and common yew (*Taxus baccata* L.) in Cádiz and Jaén provinces (Spain), respectively; in COI, the haplotype Hpa1 was the most common with twenty-nine sequences from the rhizosphere of common yew and peach at Jaén and Murcia provinces (Spain), followed by Hpa2 with three sequences from the rhizosphere of peach and apricot at Zaragoza province (Spain), and Hpa3 and Hpa4 with two sequences, both of which were from the rhizosphere of wild olive at Cádiz province (Spain) (Figure 2). In *C. plesioannuliferum*, a single haplotype was found from D2-D3 and COI sequences (HPL1 and Hpl1, respectively), both of which were from the rhizosphere of common yew at Jaén province (Spain). Additionally, in *C. annuliferum*, four haplotypes were detected for D2-D3 (HAA1, HAA2, HAA3 and HAA4, with 3, 1, 1 and 1 sequence, respectively) from grasses in Belgium (Figure 2), and two haplotypes for COI (Haa1 and Haa2), with Haa1 with eight sequences from grasses in Ireland and Belgium, and Haa2 with a single sequence from grasses in Belgium (Figure 2).

### 2.5. Systematics

#### Description of *Criconema pseudoannuliferum* sp. nov.

**Figure 3:**
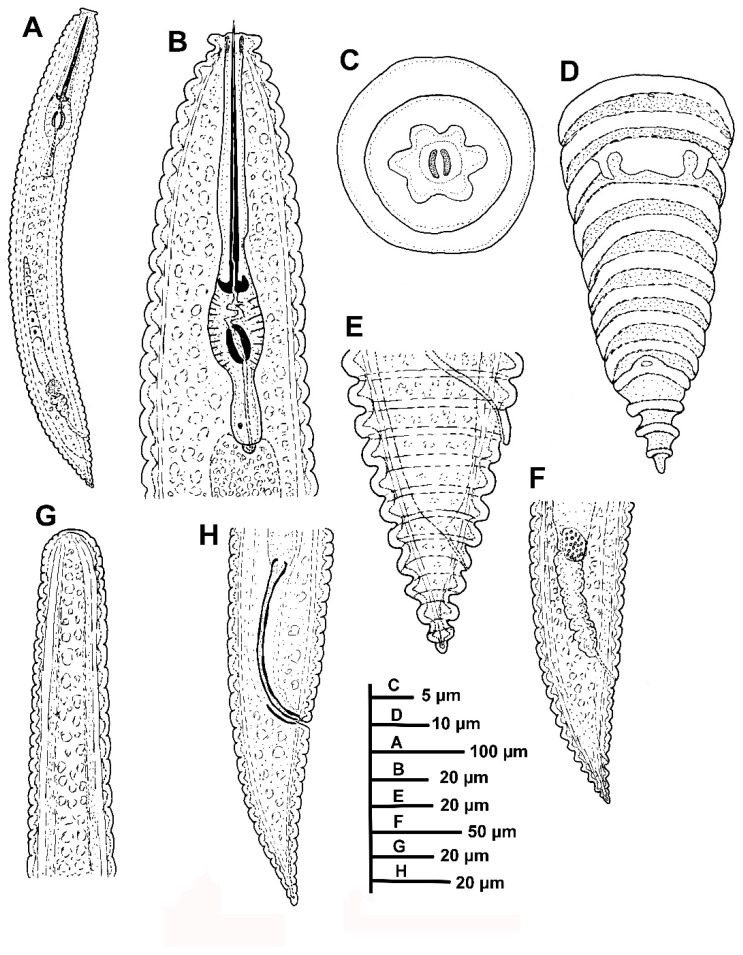
Line drawings of *Criconema pseudoannuliferum* sp. nov. (**A**), whole female, (**B**) female anterior region; (**C**), detail of *en face* view; (**D**), female posterior region in frontal view showing vulva and anus, (**E**,**F**) female posterior region; (**G**), male anterior region showing absence of stylet; (**H**), male posterior region.

**Figure 4:**
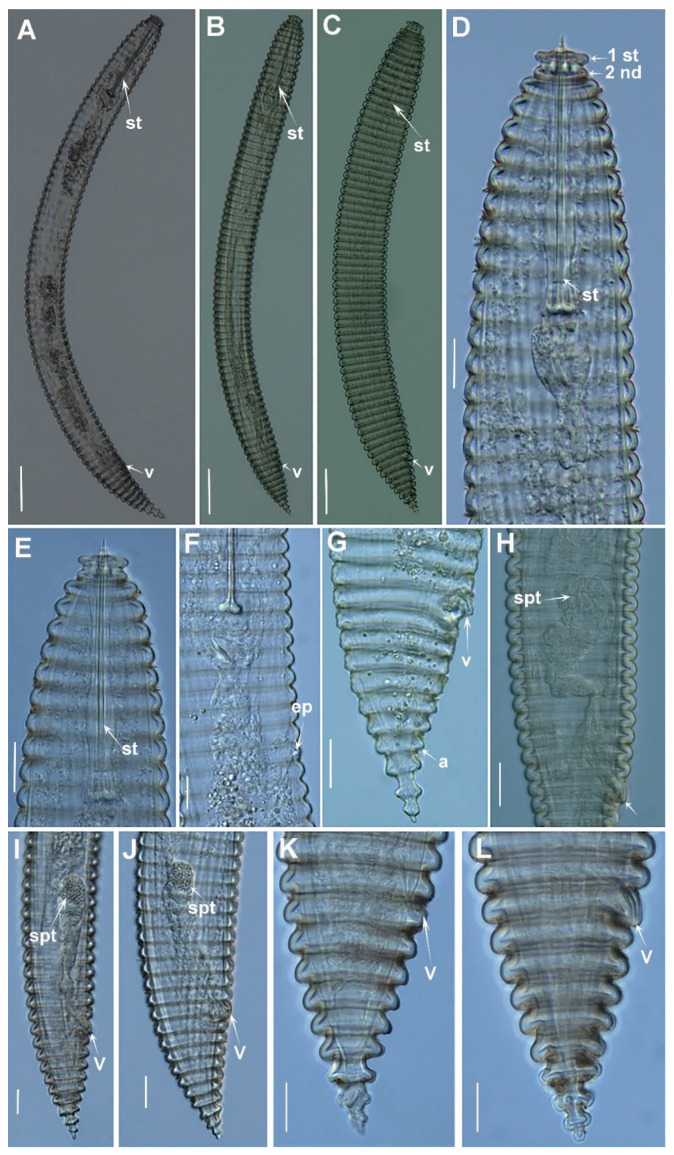
Light micrographs of *Criconema pseudoannuliferum* sp. nov. females. (**A**–**C**), whole female; (**D**), female pharyngeal region 1st and 2nd body annuli (arrowed); (**E**,**F**) female anterior region showing excretory pore; (**G**–**L**), female posterior region showing spermatheca filled with sperm (arrowed). Abbreviations: ep = excretory pore; spt = spermatheca, st = stylet; V = vulva; 1st, 2nd = first- and second-body annuli. Scale bars: (**A**–**C**) = 50 µm; (**D**–**L**) = 20 µm.

**Figure 5:**
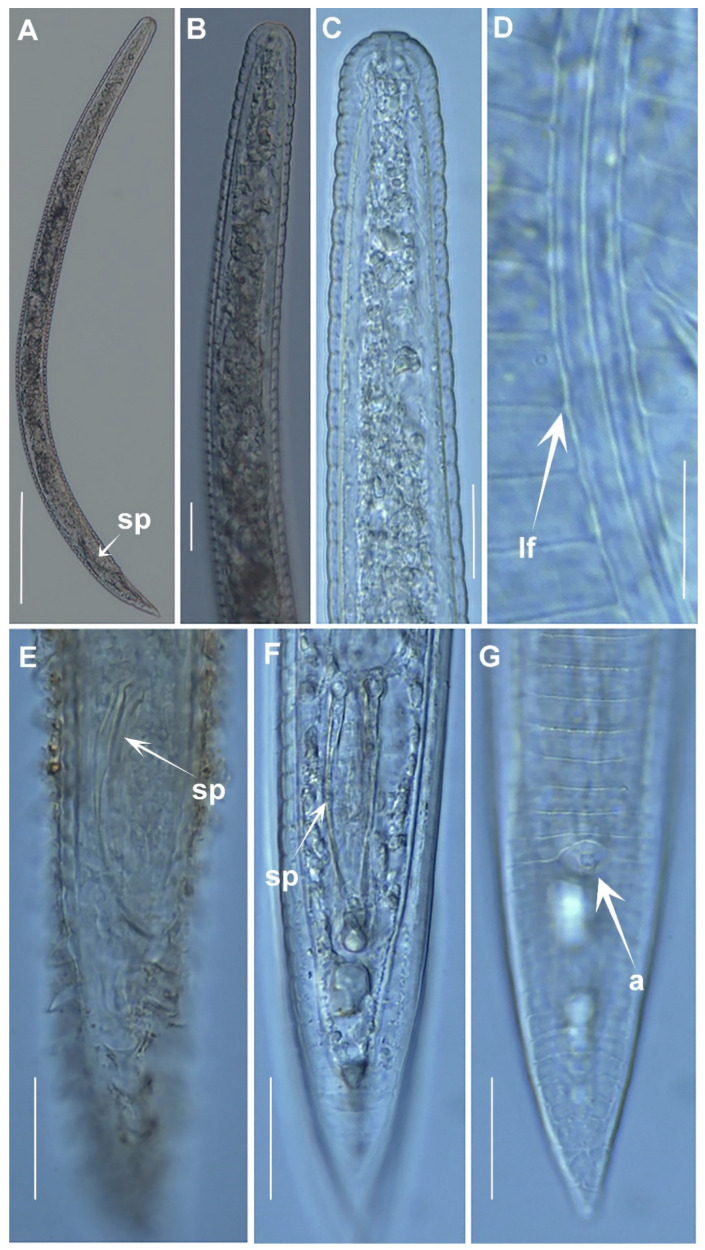
Light micrographs of *Criconema pseudoannuliferum* sp. nov. male. (**A**), whole male, showing spicules (arrowed); (**B**,**C**), male anterior region showing absence of stylet and undifferentiated pharynx. (**D**), male at mid-body showing detail of lateral field (arrowed); (**E**–**G**), male tail showing spicules and anus (arrowed). Abbreviations: a = anus; lf = lateral field; sp = spicules. Scale bars: (**A**) = 100 µm; (**B**–**G**) = 20 µm.

**Figure 6:**
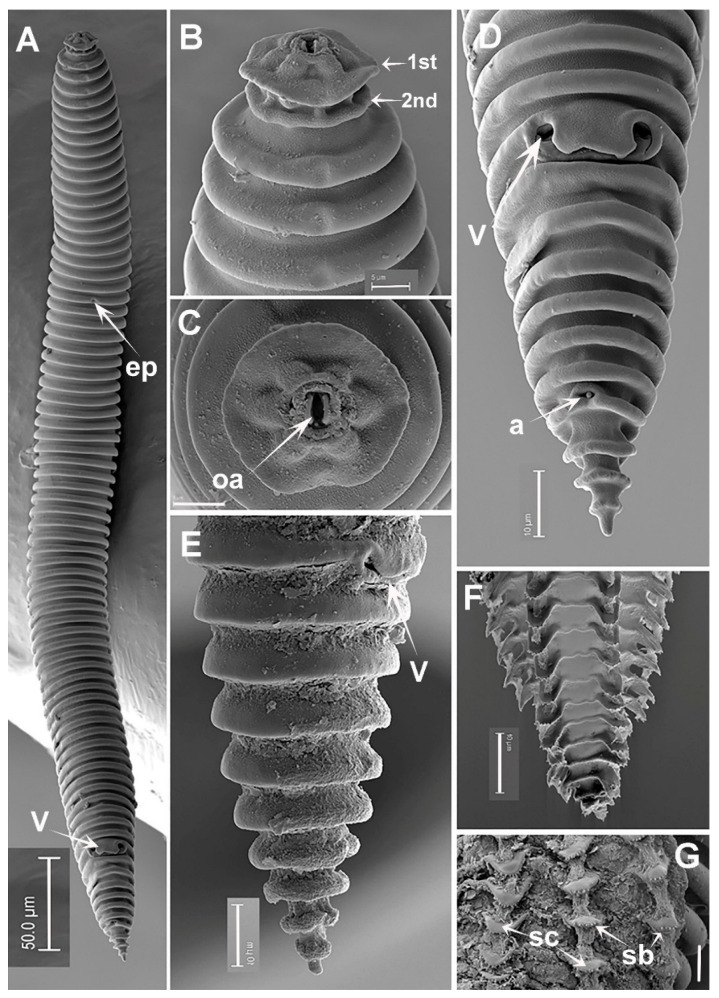
SEM micrographs of *Criconema pseudoannuliferum* sp. nov. female and fourth-juvenile stage. (**A**), whole female; (**B**), female detail of 1st and 2nd body annuli; (**C**), female en face view showing oral aperture (arrowed); (**D**,**E**), female posterior region in lateral and frontal views showing vulva and anus (arrowed); (**F**,**G**), posterior region of fourth-juvenile stage showing files of scales (arrowed) and minute short bristles (arrowed). Abbreviations: a = anus; ep = excretory pore; oa = oral aperture; sb = short bristles; sc = scales; V = vulva; 1st, 2nd = first- and second-body annuli. Scale bars: (**A**) = 50 µm; (**B**,**C**) = 5 µm; (**D**–**F**) = 10 µm; (**G**) = 5 µm.

(Figure 3, Figure 4, Figure 5 and Figure 6, Table 3). http://zoobank.org/urn:lsid:zoobank.org:act:F2E06A4F-4366-4528-A508-1845A8630180 (accessed on 18 November 2022).

*Female*: The body slightly arcuated ventrally after heat-fixing, a broad body annuli 9.0–10.0 µm wide was observed, rounded with smooth edges and without anastomoses. The lip region was small, with two annuli, the first annulus wider than second, anteriorly directed, and the second annulus making a collar-like appearance. SEM pictures displayed an elongate oral aperture, without submedian lobes, and six distinct hexaradiate pseudolips. The stylet was long, commonly straight but rarely slightly curved in few specimens, constituting 17.4 (15.0–20.9)% of body length or 669. (62.0–72.4)% of pharynx length, the knobs were anchor-shaped, 11.1 (10.0–13.0) µm wide and 3.3 (3.0–4.0) µm high. The dorsal pharyngeal gland opening was 10.2 ± 1.0 (8.0–12.0) µm from base of stylet. The nerve ring surrounding isthmus was situated at 136 (125–140) µm from anterior end. The excretory pore was usually 1–2 annuli back of pharyngeal base, 163–191 µm from the anterior end. Hemizonid was not detected. A reproductive system was observed. It included being monodelphic-prodelphic, outstretched (228–486 µm long), and it composed of a long ovary with oocytes arranged in one single row. Spermatheca were well developed, round to oval [21.5 ± 1.8 (18.0–23.0) µm, 15.8 ± 1.6 (13.0–18.0) µm] and filled with rounded sperm (ca. 1 µm wide). The vulva closed, the anterior lip was not overhanging posterior one, the vagina was straight, the vulva-anus distance was 1.2–2.3 times of tail length. The anus was hardly visible, the tail was rudely arrowing to a conical shape, with 4–6 annuli, and the terminal annulus was usually not lobed.

*Male*: The male was infrequent (1 male: 10 females), only two specimens were detected. One of them was detected inside the cuticle of fourth-stage juvenile and was measured and sequenced. The body was slightly curved ventrally, narrowing to the tail region. The lip region was rounded, almost hemispherical, the stylet was absent, the pharynx was undifferentiated and not functional, lateral fields with three incisures observed. The testis was straight and it was 28.6, 37.0 % of total body length. The tail conoid was observed with a finely rounded terminus, the bursa was absent, the spicules were cephalated, slender and ventrally curved, the gubernaculum was simple and slightly curved ventrally.

*Juveniles*: The body a similar morphological appearance to the female, but it showed annuli margins with eight to ten rows of projections or scales in all the annuli along the body. The lip region was observed with collar-like first annulus. Borders of projections or scales with a row of six to eight short bristles only differentiated under SEM analysis.

##### Diagnosis and Relationships

*Criconema pseudoannuliferum* sp. nov. is characterized by the following measurements and observations: a medium-large-sized female body 526–715 µm, stylet = 105.0–116.0 µm, V = 87.7–91.7, c = 19.6–29.5, c′ = 1.0–1.4, R = 67–79, RV = 9–11, Ran = 4–6, VL/VB = 1.3–1.9, tail rudely arrowing to a conical shape, terminal annulus usually not lobed, and males rarely without a bursa and spicules 47.0–48.0 µm.

According to a dichotomous key by Geraert [2], the new population of *Criconema* from Casares, Malaga, is morphologically very close to *C. annuliferum* and *C. plesioannuliferum* and *C. paraannuliferum* [2,11,13]. The morphological and morphometrical data from *C*. *pseudoannuliferum* sp. nov. are within the range of the original description of *C. plesioannuliferum*, *C. paraannuliferum* and *C. annuliferum*, as well as reports by Peneva et al. [40] from oak forests in Russia, and Etongwe et al. [11] from Belgium, and the slight differences detected were within the range of intraspecific variation. In addition, the new species can be separated from *C. plesioannuliferum*, *C. paraannuliferum* and *C. annuliferum* by spicule length (47.0–48.0 vs. 29.0–32.0, 30.5–37.0, 51–53 µm, respectively) [11,13]. However, molecular characterization by ribosomal and mitochondrial genes clearly separated *C*. *pseudoannuliferum* sp. nov. from *C. plesioannuliferum*, *C. paraannuliferum* and *C. annuliferum*. *C. crotaloides* can be differentiated by RV (9–11 annuli from terminus vs. 11–14), and Ran (4–6 annuli from terminus vs. 6–9) [15,41]. Finally, *C. iranicum* can be separated by a longer stylet (105.0–116.0 vs. 76.5–84.0 µm) [42].

##### Molecular Characterization

Eight D2-D3 of the 28S rRNA (ON877364-ON877371), six ITS (ON877372-ON877377), eight 18S (ON877378-ON877385), and eleven COI gene sequences (ON899944-ON899954) were generated for this new species. No intraspecific variation was detected for D2-D3, ITS, and 18S, and 98.7–100.0% intraspecific variability (zero to eight nucleotides, zero indels) for COI. The closest species to *C. pseudoannuliferum* sp. nov. for the D2-D3 region was *C. annuliferum* (MN786697-MN783702) from Belgium [11], being 96.6% similar (differing thirty-two nucleotides, zero indels), followed by *C. paraannuliferum* being 95.2% similar (ON705053-ON705072) (differing thirty-two to thirty-three nucleotides, zero indels), *C. plesioannuliferum* being 94.6% similar (ON705073-ON705080) (differing thirty-six nucleotides, zero indels) from Spain [13], and 94.1% similar to *C. demani* (MN628432) (differing thirty-eight nucleotides, one indel) from Iceland [43]. ITS region was only 86.20% similar to *C. paraannuliferum* (ON705082-ON705102) (differing 87–95 nucleotides, 23 indels), 85.88% similar to *C. plesioannuliferum* (ON705103-ON705116) (differing 105–108 nucleotides, 27 indels) from Spain [13] and 81.68% similar to *C. silvum* (MF683236-MF683237) (differing in 140–141 nucleotides, 41 indels) from South Africa [44]. The 18S rRNA showed high similarity values: they were 99.59% similar to *C. petasum* (MF094927) (differing seven nucleotides, zero indels) from the USA [10], 99.59% similar to *Criconema* sp. PDL-2005 (AJ966480) (differing seven nucleotides, zero indels) from Belgium [45], 99.29% similar to *C. paraannuliferum* (ON705040-ON705042) (differing twelve nucleotides, zero indels), 99.24–99.29% similar to *C. plesioannuliferum* (ON705048-ON705050) (differing thirteen to fourteen nucleotides, zero to one indels) from Spain [13], and 99.29% similar to *Ogma octangularis* (MF094956) (differing twelve nucleotides, zero indels) from the USA [10]. For COI gene sequences (ON899944-ON899954), the similarity values were 86.62–86.73% (differing 93 nucleotides, no indels) from *C. plesioannuliferum* from Spain [13], 86.38–87.12% similar to *C. loofi* (KX290561-KX290566) (differing 88–93 nucleotides, no indels) from the USA [46], 86.40–86.62% (differing 92–97 nucleotides, no indels) from *C. paraannuliferum* from Spain [13], and 86.53% similar to *Crossonema menzeli* (MN710938) (differing 92 nucleotides, no indels) from Denmark [12].

##### Type Habitat and Locality

*Criconema pseudoannuliferum* sp. nov. was found in the rhizosphere of maritime pine (*Pinus pinaster* Ait.) (coordinates 36°28′55.1″ N, 5°14′37.1″ W); the municipal district of Casares, in Bermeja-Crestellina Mountain, Málaga province, south-eastern Spain.

##### Etymology

The species epithet, *pseudoannuliferum*, refers to Greek *pseudo*—meaning “in appearance only, resembling” because of its close resemblance to *Criconema annuliferum*.

##### Type Material

A holotype female, twenty paratypes females and four fourth-stage juveniles paratypes (slide numbers WPPp3-06, WPPp3-01–WPPp3-05, WPPp3-8–WPPp3-11) were deposited in the Nematode Collection of the Institute for Sustainable Agriculture, CSIC, Córdoba, Spain; two females at Istituto per la Protezione delle Piante (IPP) of Consiglio Nazionale delle Ricerche (C.N.R.), Sezione di Bari, Bari, Italy (WPPp3-12); and two females were deposited at the USDA Nematode Collection (slide T-7738t).

### 2.6. Phylogenetic Analyses of Criconema annuliferum Species Complex

**Figure 7:**
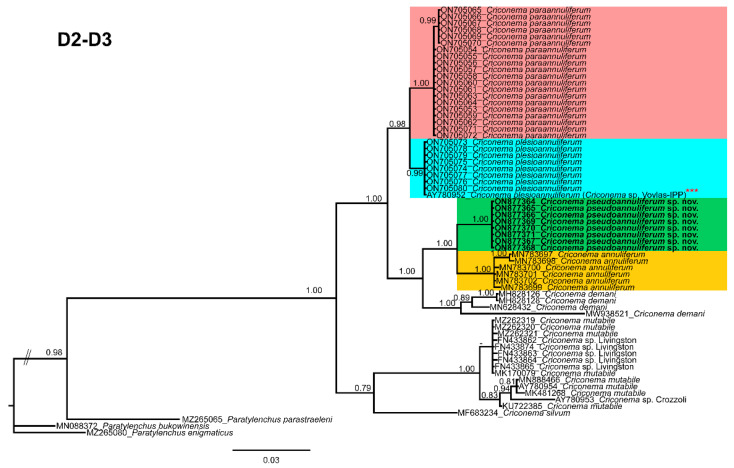
Phylogenetic relationships within the genus *Criconema*. Bayesian 50% majority rule consensus tree as inferred from D2-D3 expansion domains of the 28S rRNA sequence alignment under transition model with invariable sites and a gamma-shaped distribution (TIM3 + I + G). Posterior probabilities of more than 0.70 are given for appropriate clades. Newly obtained sequences in this study are shown in bold. The scale bar indicates expected changes per site, and the coloured boxes indicate the clade association of *Criconema annuliferum* complex. *** Initially identified in NCBI as *Criconema* sp. Vovlas-Italy.

**Figure 8:**
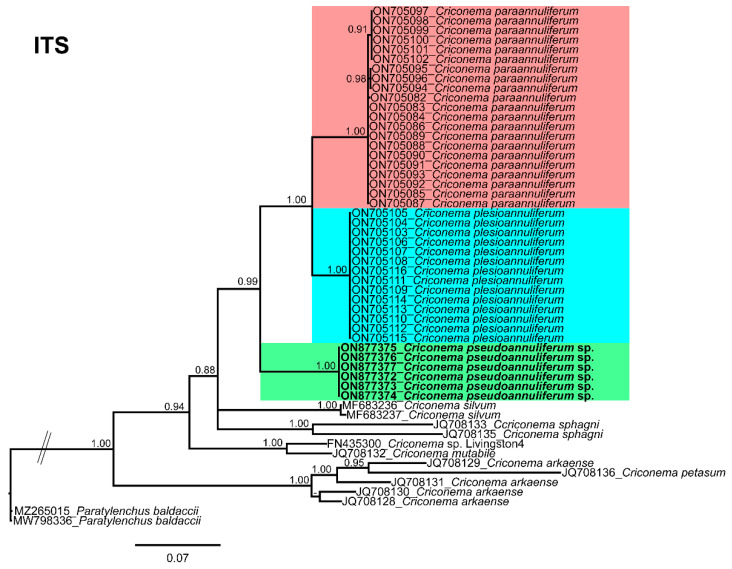
Phylogenetic relationships within the genus *Criconema*. Bayesian 50% majority rule consensus tree as inferred from ITS rRNA sequence alignment under the transversion model with invariable sites and a gamma-shaped distribution (TVM + I + G). Posterior probabilities of more than 0.70 are given for appropriate clades. Newly obtained sequences in this study are shown in bold. The scale bar indicates expected changes per site, and the coloured boxes indicate the clade association of *Criconema annuliferum* complex.

**Figure 9:**
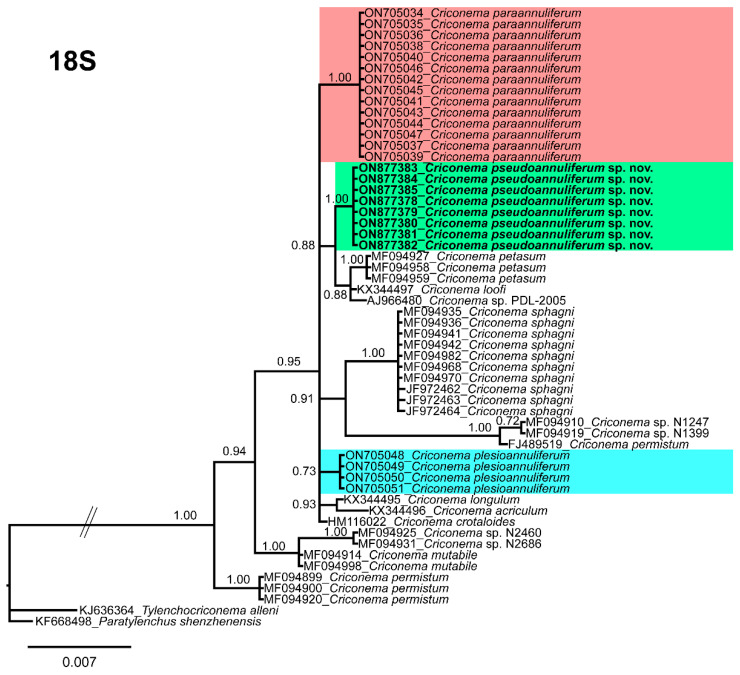
Phylogenetic relationships within the genus *Criconema*. Bayesian 50% majority rule consensus tree as inferred from 18S rRNA sequence alignment under the one-parameter model with invariable sites and gamma distribution model (TPM3uf + I + G). Posterior probabilities of more than 0.70 are given for appropriate clades. Newly obtained sequences in this study are shown in bold. The scale bar indicates expected changes per site, and the coloured boxes indicate the clade association of *Criconema annuliferum* complex.

**Figure 10:**
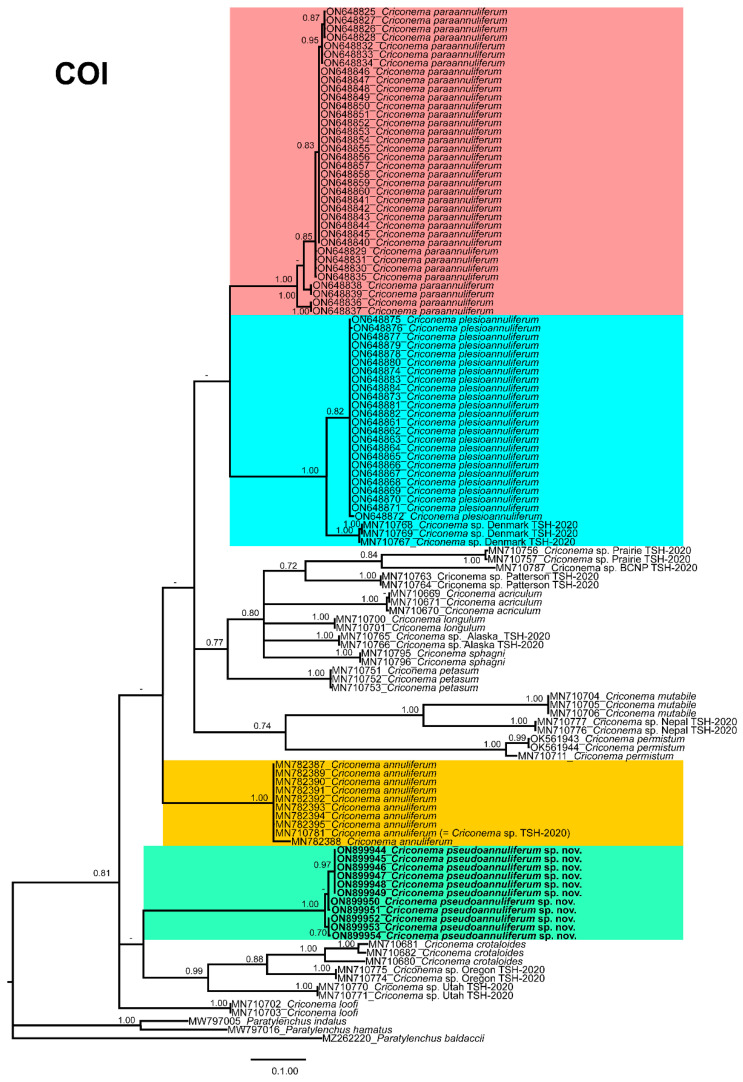
Phylogenetic relationships within the genus *Criconema*. Bayesian 50% majority rule consensus tree as inferred from cytochrome c oxidase subunit 1 (COI) sequence alignment under the transition model with invariable sites and a gamma-shaped distribution (TIM2 + I + G). Posterior probabilities of more than 0.70 are given for appropriate clades. Newly obtained sequences in this study are shown in bold. The scale bar indicates expected changes per site, and the coloured boxes indicate the clade association of *Criconema annuliferum* complex. *Criconema* sp. Ireland TSH2020 should be considered *C. annuliferum*.

Phylogenetic relationships among *C. annuliferum* species complex completed from analyses of D2-D3 expansion segments of 28S rRNA, ITS rRNA, 18S rRNA, and COI mtDNA fragments using Bayesian inference (BI) are given in Figure 7, Figure 8, Figure 9 and Figure 10, respectively. The D2-D3 domains of the 28S rRNA gene alignment (698 bp long) included ninety-six sequences of thirty-nine Criconematidae species and three outgroup species (*Paratylenchus bukowinensis* (MN088372), *Paratylenchus enigmaticus* (MZ265080) and *Paratylenchus parastraeleni* (MZ265065)). Eight new sequences were included in this analysis. The Bayesian 50% majority rule consensus tree inferred from the D2-D3 alignment is given in Figure 7. For this marker, species belonging to the species complex *C. annuliferum* clustered in two well-supported (PP = 0.96, 1.00, respectively) separated clades (Figure 7), each one subdivided into two subclades, one of them (PP = 1.00) comprised *C. paraannuliferum* (ON705053-ON705072) and *C. plesioannuliferum* (ON705073-ON705080) (PP = 0.98) from Spain [13], and the other one (PP = 1.00) by *C. pseudoannuliferum* sp. nov. (ON877364-ON877371) and *C. annuliferum* (MN783697-MN783702) (PP = 1.00) from Belgium [11] (Figure 7). In this analysis, we detected that the genus *Criconema* was not a monophyletic group within Criconematidae, appearing in four different clades, although some clades or subclades were not well-supported (Figure 7).

The ITS rRNA gene alignment (750 bp) included 75 sequences of 34 Criconematidae species and two outgroup sequences of *Paratylenchus baldaccii* (MW798336, MZ265015). Six new sequences were included in this analysis. The Bayesian 50% majority rule consensus tree inferred from the ITS alignment is given in Figure 8. The tree showed a well-supported subclade (PP = 1.00) with *C. paraannuliferum* (ON705081-ON705102) and *C. plesioannuliferum* (ON705103-ON705116), clustering with the subclade of *C. pseudoannuliferum* sp. nov. (ON877372-ON877377) in a low supported clade (PP = 0.57), but clearly separated from *C. paraannuliferum* and *C. plesioannuliferum* from Spain [13] (Figure 8). Additionally, in this analysis, we detected that the genus *Criconema* was not a monophyletic group within Criconematidae, appearing in five different clades, although some clades or subclades were not well-supported (Figure 8).

The 18S rRNA gene alignment (1689 bp long) included 89 sequences of 47 Criconematidae species and two outgroup species (*Tylenchocriconema alleni* (KJ636364) and *Paratylenchus shenzhenensis* (KF668498)). Eight new sequences were included in this analysis. The Bayesian 50% majority rule consensus tree inferred from the 18S rRNA sequence alignment is given in Figure 9. The 18S tree showed that *C. pseudoannuliferum* sp. nov. clustered in a poorly supported (PP = 0.66) subclade together with *C. petasum*, *Criconema* sp. PDL-2005 and *C. loofi* from the USA [10], Belgium [45], and from the USA [10], respectively (Figure 9). Additionally, in this analysis, we detected that the genus *Criconema* was not a monophyletic group within Criconematidae, appearing in ten different clades, although several clades or subclades were not well-supported (Figure 9).

The COI gene alignment (714 bp long) included 142 sequences of 39 Criconematidae species and three outgroup species: *Paratylenchus baldaccii* (MZ262220), *Paratylenchus hamatus* (MW797016) and *Paratylenchus indalus* (MW797005). Eleven new sequences were included in this analysis. The Bayesian 50% majority rule consensus tree inferred from the COI sequence alignment which is given in Figure 10. The COI tree showed that *C. pseudoannuliferum* sp. nov. clustered in a low supported (PP = 0.75) subclade together with *C. crotaloides*, *Criconema* sp. Oregon TSH-2020 and *Criconema* sp. Utah TSH-2020, all of them from the USA [12] (Figure 9). Additionally, in this analysis, we detected that the genus *Criconema* was not a monophyletic group within Criconematidae, appearing in ten different clades, although several clades or subclades were not well-supported (Figure 9).

## 3. Discussion

The primary objective of this study was to clarify the identity of a morphospecies population resembling the *Criconema annuliferum* species complex associated with maritime pine (*Pinus pinaster* Ait.) in a mountainous area from the Málaga province in southern Spain by means of integrative taxonomical approaches (morphological, morphometrical and molecular). Our results from the blind-identification test demonstrated that morphological and morphometrical analyses identified this species as belonging to the *C. annuliferum* species complex, but former analyses identified a priori as *C. plesioannuliferum*, and the latter as mixed populations of *C. paraannuliferum* and *C. plesioannuliferum*. However, anatomo-morphometric studies integrated with ribosomal DNA and mitochondrial DNA molecular markers (D2-D3 expansion domains of the 28S rRNA gene, ITS rRNA gene and the mtDNA gene COI) clearly revealed that this population from maritime pine represent a new linage within the *C. annuliferum* species complex, clearly independent from *C. paraannuliferum* and *C. plesioannuliferum*, and related only to *C. annuliferum* from Belgium in the D2-D3 phylogenetic analysis. These results supported a general hypothesis that hyper-cryptic species complexes are likely to be common in several organismal groups, including ring nematodes of the genus *Criconema*. Although it is difficult to explain why hyper-cryptic species complex are particularly common in some taxa, ecological differences between habitats may affect rates of diversification, as hypothesised by Pérez-Ponce de León & Poulin [47]. In our case, *C. paraannuliferum* was mostly associated with cultivated crops in several localities in Spain, whereas *C. plesioannuliferum* was associated with particular cool and dry environmental conditions of a common yew forest in a mountainous area at Jaén province, and *C. pseudoannuliferum* sp. nov. under a relative mild and humid climate in a maritime pine forest in a mountainous area of Málaga province, both in southern Spain [13]. Our results validating that accurate identification and naming of newly found cryptic species in the *C. annuliferum* species complex and other plant-parasitic nematodes is fundamental for their subsequent routine recognition and to realise representative estimates of real biodiversity [48]. Ribosomal and mitochondrial markers (D2-D3 expansion domains of the 28S rRNA gene, ITS rRNA gene and the mtDNA gene COI) are important tools for accurate identification of *Criconema* spp. In the present study, remarkably, all four molecular markers clearly separated *C. pseudoannuliferum* sp. nov. from the closet species in the *C. annuliferum* species complex, supporting that this group is a case of hyper-cryptic species complex comprised by the new species together with *C. annuliferum*, *C. paraannuliferum* and *C. plesioannuliferum*. Sequence divergence at COI was higher than that for D2-D3 and ITS markers most probably, because mtDNA accumulated nucleotide substitutions at a much higher rate of substitutions than D2-D3 and ITS, as occurred in other animal groups [49]. The present results confirm also that cryptic speciation in criconematids may be more common than previously expected; therefore, these data supported the hypothesis that criconematids nematodes in general and *C. annuliferum* species complex in particular are a hyperdiverse group of organisms [26,50,51].

Multivariate morphometric analyses have been recognised as useful tools for species separation in plant-parasitic nematodes, especially for those of special phytopathological interest belonging to the genus *Xiphinema* [32,52,53], the genus *Longidorus* [32,54] and *Criconema* [10]. Our data support that the *C. annuliferum* complex comprises a model example of morphostatic speciation because independent approaches based on multivariate morphometric and molecular analyses clearly separated four species within this cryptic complex [55]. The results of the multivariate analysis supported the overall shape and size of the nematode body, especially the posterior section of the nematode, and key morphometric character to differentiate and identify species within the genus *Criconema* [2]. However, it should be emphasized that this was only valuable to differentiate some closely related species within the *C. annuliferum* complex (Table 1, Figure 1), in particular *C. plesioannuliferum*, as previously described by Clavero-Camacho et al. [13]. In this study, multivariate analysis also supported the idea that *C. pseudoannuliferum* sp. nov., *C. paraannuliferum* and *C. annuliferum* could resemble the same species (Figure 1), since they shared similar values for the most of key diagnostic characters in the genus *Criconema*, making their accurate identification difficult and increasing the level of complexity within this cryptic species complex.

The TCS haplotype analysis inferred from ribosomal and mitochondrial genes showed four well-differentiated haplogroups corresponding to four different main lineages (*C. pseudoannuliferum* sp. nov., *C. paraannuliferum*, *C. plesioannuliferum* and *C. annuliferum*). Overall haplotype diversity within *C. annuliferum* species complex was higher in mitochondrial than in ribosomal gene. showing that *C. paraannuliferum* and *C. pseudoannuliferum* sp. nov. displayed the same molecular diversity in COI with four haplotypes, but in the former, these differences appeared to be related to the geographical origin of the studied populations, but in the latter, all the specimens were located within the same locality. However, low diversity was detected in COI within *C. annuliferum* (apparently irrespective of geographic origin, Belgium or Ireland) and no diversity within *C. plesioannuliferum*, from which all specimens were located in the same locality and environmental conditions (common yew forest). The higher variability differences in COI can be related, unlike intraspecific mitogenomic plasticity due to nucleotide mutation rates by replication errors or with reproductively isolated populations under particular environmental conditions [56]. These data agree with other occurring situations in criconematids [46], and also concur with the hypothesis of faster coalescence within species linages in mitochondrial than nuclear markers [57,58,59].

Phylogenetic analyses based on D2-D3, ITS, 18S and COI gene using BI mostly agreed with the clustering obtained by other authors [8,11,46,60]. Ribosomal and mitochondrial phylogenies clearly separated the *C. annuliferum* complex in four separate species, which was also confirmed by morphometric and molecular species delimitation analyses. The phylogenetic positions of several Criconematidae genera based on ribosomal and mitochondrial DNA markers were not well-resolved, and the majority of them, including the genus *Criconema*, did not show monophyly, appearing in different clades, which agreed with previous studies [10,11,61]. Further researches are needed within this complex (wide-phylogeographical studies) to confirm if the four separated lineages within the *C. annuliferum* species complex are the result of an allopatric or sympatric model of speciation [62].

Finally, the current results highlighted previous results on the noteworthy biodiversity of several groups of plant-parasitic nematodes in southern Spain, such as species within the Longidoridae family (including virus vector nematodes of the genera *Xiphinema* and *Longidorus*), pin nematodes of the genus *Paratylenchus* [13,33,54,63], or *Criconema* [13]. The results warrant additional sampling efforts to clarify the real biodiversity in Spain.

## 4. Materials and Methods

### 4.1. Sampling Sites and Nematode Morphological Identification

Ten soil samples were collected from the rhizosphere of maritime pine (*Pinus pinaster* Ait.) forests in the Bermeja-Crestellina mountains, located in the western part of Málaga province, belonging to the municipality of Casares, in Málaga province, southern Spain. Samples were collected within 5–40 cm soil depth. Nematodes were extracted from a 500-cm^3^ subsample of soil by centrifugal flotation [64].

A total of 27 specimens comprising 21 females, 2 males and 4 juveniles were studied for morphological and morphometrical analyses. Individuals for light microscopy (LM) analysis were killed and fixed in an aqueous solution of 4% formaldehyde + 1% glycerol, dehydrated using alcohol-saturated chamber and processed to pure glycerine using Seinhorst’s method [65], as modified by De Grisse [66]. Light micrographs were taken using alive immobilized nematodes and measurements of each nematode population, including significant diagnostic characteristics (i.e., de Man indices, body length, stylet length, lip region, tail shape) [35] were completed using a Leica DM6 compound microscope with a Leica DFC7000 T digital camera (Wetzlar, Germany) using fixed and mounted nematodes in glycerine. Nematodes were identified at the species level applying an integrative approach merging morphological techniques and molecular analyses to achieve an efficient and accurate identification [13,35]. Fixed mounted individuals were examined and measurements of each nematode included important diagnosis characteristics as suggested in previous studies [13,35], and the sequencing of specific molecular markers (listed below) corroborated the identity of the nematode species for individual specimens.

Females and fourth-stage juveniles of this species mounted in glycerine were selected for SEM observations. The nematodes were hydrated in distilled water, dehydrated in a graded ethanol-acetone series, critical point-dried, coated with gold, and observed with a Zeiss Merlin scanning electron microscope (5 kV) (Zeiss, Oberkochen, Germany) [67].

Voucher specimens of the described species were deposited in the nematode collection of the Institute for Sustainable Agriculture, IAS-CSIC, Córdoba, Spain.

### 4.2. Blind-Identification Test

Fixed mounted nematodes in glycerine were consecutively distributed among different members of the nematological team (who previously described *C. paraannuliferum* and *C. plesioannuliferum*) for morphological and morphometrical identification, but without giving any data on the molecular identity of the unknown *Criconema* sp. population. The nematological team was divided in three separate groups comprising IAS-CSIC group (Institute for Sustainable Agriculture-Spanish National Research Council), IFAPA group (Andalusian Institute of Agricultural and Fisheries Research and Training) and UJA group (Department of Animal Biology, University of Jaén). Each laboratory studied the complete set of *Criconema* slide samples from maritime pine, but without knowing a priori the results on molecular analyses. IAS-CSIC laboratory started measuring all the specimens, including 21 females, 2 males and 4 juveniles, that were incorporated to this manuscript and were shared with the rest of laboratories for the blind-identification test.

### 4.3. DNA Extraction, PCR and Sequencing

DNA extraction was always based on single nematode specimens as defined by Palomares-Rius et al. [68], and more decisive, for all studied specimens, all the four molecular markers studied of each individual originated from the same single DNA-extracted nematode in each PCR tube without any exception. Furthermore, assignation of male and juvenile stages always was proven by single DNA extraction of these individuals. Additionally, for avoiding mistakes, in case of mixed *Criconema* populations within the same soil sample (as occurred in a previous study on *Criconema*), single nematodes were provisionally deposited in a drop of 1 M NaCl containing glass beads (to avoid nematode crushing/damaging specimens) to ensure specimens conformed with the unidentified population. Briefly, an individual nematode was cut using a scalpel in a drop of PCR buffer (ThermoPol^®^, Biolabs, New England, USA) (20 µL) and 2 μL proteinase K (600 μg/mL) were added. The tubes were frozen at −80 °C (15 min), then incubated at 65 °C (1 h) and at 95 °C (10 min) consecutively. Tubes were centrifuged (1 min, 16,000× *g*) and kept at −20 °C until used in PCR; and more importantly, all the four molecular markers of each *Criconema* belonged to the same single extracted individual in each PCR tube without any exception.

The D2 and D3 expansion domains of the 28S rRNA were amplified using the D2A (5′-ACAAGTACCGTGAGGGAAAGTTG-3′) and D3B (5′-TCGGAAGGAACCAGCTACTA-3′) primers [69]. The Internal Transcribed Spacer region (ITS) was amplified by using forward primer TW81 (5′-GTTTCCGTAGGTGAACCTGC-3′) and reverse primer AB28 (5′-ATATGCTTAAGTTCAGCGGGT-3′) [70]. The partial 18S rRNA was amplified using the primers 988 (5′-CTCAAAGATTAAGCCATGC-3′) and 1912R (5′-TTTACGGTCAGAACTAGGG-3′) [Holterman et al., 2006]. Additionally, the COI gene was amplified using the primers COI-F5 (5′-AATWTWGGTGTTGGAACTTCTTGAAC-3′ and COI-R9 (5′-CTTAAAACATAATGRAAATGWGCWACWACATAATAAGTATC-3′) [71]. The PCR cycling conditions for the 28S rRNA, ITS and 18S rRNA were as follows: 95 °C for 15 min, followed by 35 cycles of 94 °C for 30 s, an annealing temperature of 55 °C for 45 s, and 72 °C for 1 min, and one final cycle of 72 °C for 10 min. The PCR cycling for COI primers was as follows: 95 °C for 15 min, 39 cycles at 94 °C for 30 s, 53 °C for 30 s, and 68 °C for 1 min, followed by a final extension at 72 °C for 7 min. PCR volumes were adapted to 25 µL for each reaction, and primer concentrations were as described in De Ley et al. [69], Subbotin et al. [8], Holterman et al. [72] and Powers et al. [71]. We used 5× HOT FIREpol Blend Master Mix (Solis Biodyne, Tartu, Estonia) in all PCR reactions. The PCR products were purified using ExoSAP-IT (Affimetrix, USB products, Kandel, Germany) and used for direct sequencing in both directions with the corresponding primers. The resulting products were run in a DNA multicapillary sequencer (Model 3130XL Genetic Analyzer; Applied Biosystems, Foster City, CA, USA), using the BigDye Terminator Sequencing Kit v.3.1 (Applied Bio-systems, Waltham, MA, USA) at the Stab Vida sequencing facility (Caparica, Portugal). The sequence chromatograms of the four markers (18S, ITS, COI and D2-D3 expansion segments of 28S rRNA) were analysed using DNASTAR LASERGENE SeqMan v. 7.1.0. Basic local alignment search tool (BLAST) at the National Center for Biotechnology Information (NCBI) was used to confirm the species identity of the DNA sequences obtained in this study [73]. The newly obtained sequences were deposited in the GenBank database under accession numbers indicated on the phylogenetic trees.

### 4.4. Species Delimitation within Criconema annuliferum Species Complex

Two different approaches of species delimitation were used to determine species boundaries within this *C. annuliferum* species complex including morphometric and molecular data. *Criconema annuliferum* species complex can be recognized morphologically by the lip region shape composed by two annuli being the first wider than the second one, as well as the total number of annuli on body (R), and between posterior end of body and vulva (RV) [2]. This species complex comprises *C. annuliferum*, *C. crotaloides*, *C. paraannuliferum*, *C. plesioannuliferum* and the new taxa described here as *C. pseudoannuliferum* sp. nov. [13]. The recognition of the *C. annuliferum* species complex included key morphometric characters but also used accurate studies of integrative taxonomy *viz*. availability of molecular markers linked to accurate morphology identification by morphometry in order to avoid misidentifications. Because of the lack of integrative taxonomical data on *C. crotaloides*, this species could not be included in the previous or present study [12,13].

Species delineation using morphometry was conducted using factor analysis (FA) [74], and following the procedure described by Clavero-Camacho et al. [13]. A new ring nematode population from the rhizosphere of maritime pine (*Pinus pinaster* Ait.) in Casares, Málaga province, southern Spain, was added to the previous morphometric analysis in the *C. annuliferum* species complex [13]. Overall, 106 female specimens were used in multivariate approach for *C. annuliferum* complex. Prior to the statistical analysis, key diagnostic characters were tested for collinearity [75,76]. We used the collinearity test based on the values of the variance inflation factor (VIF) method that iteratively excluded numeric covariates showing VIF values > 10 as suggested by Montgomery et al. [77]. FA was performed using maximum likelihood algorithm through a decomposition of the data matrix between populations using the fa function implemented in the package ‘psych’ [78]. Finally, a minimum spanning tree (MST) based on the Euclidean distance was superimposed on the scatter plot of the *C. annuliferum*-specimens complex against the FA axes. MST was performed using the ComputeMST function implemented in the package ‘emstreeR’ [79]. All statistical analyses were performed using the R v. 3.5.1 freeware [80].

Species demarcation was also based on molecular data achieved by using species delimitation plugin [36] from the program Geneious Prime v2022.1.1. (Geneious, Auckland, New Zealand), and was applied to compute intra- and inter-species variation by means of the P ID liberal and the Rosenberg’s P_AB_ value. The intra- and inter-species molecular variation was established by determining the ratio between the average genetic distance between specimens within a species and the average genetic distance between specimens belonging to sister species (the average pairwise tree distance among members of a putative species/the average pairwise tree distance between the members of one putative species and the members of the closest second putative species). If ratio was less than 0.10, the probability of species identification was high [36]. The P ID (Liberal) value [38] represented the probability that a correct species identification would be made using best sequence alignment (BLAST), closest genetic distance or placement on a tree (falling within or being sister to a monophyletic species clade). Species with P ID (Liberal) ≥0.93 were considered to be adequately delimited [37]. The Rosenberg’s P_AB_ represents the probability that the monophyly of a group of sequences was the result of random branching [39].

### 4.5. Phylogenetic Analyses

D2-D3 expansion segments of the 28S rRNA, ITS rRNA, 18S rRNA and COI mtDNA fragments of the *Criconema* population from maritime pine was obtained in this study. These sequences and other sequences of Criconematidae species from GenBank were employed for phylogenetic analyses. For each dataset, the outgroup taxa selection was constructed according to previously published phylogenies and considering the molecular diversity of each dataset [11,60,61]. Multiple sequence alignments of the different genes were completed using the FFT-NS-2 algorithm of MAFFT V.7.450 [81]. BioEdit program V. 7.2.5 [82] was used for sequence alignments visualization and manually edited and trimmed of the poorly aligned positions, using a light filtering strategy (up to 20% of alignment positions), which has little impact on tree accuracy and may save computation time as suggested by Tan et al. [83], since methods for automated filtering of multiple sequence alignments frequently worsen single-gene phylogenetic inference [83]. Phylogenetic analyses of the sequence datasets were based on Bayesian inference (BI) using MrBayes 3.1.2 [84]. The best-fit model of DNA evolution was achieved using JModelTest V.2.1.7 [85] with the Akaike information criterion (AIC). The best-fit model, the base frequency, the proportion of invariable sites, the gamma distribution shape parameters and substitution rates in the AIC were then used in MrBayes for the phylogenetic analyses. The transition model with invariable sites and a gamma-shaped distribution (TIM3 + I + G, TIM2 + I + G) the D2-D3 segments of 28S rRNA and COI gene, respectively, the transversion model with invariable sites and a gamma-shaped distribution (TVM + I + G) for ITS rRNA region, and the one-parameter model with invariable sites and gamma distribution model (TPM3uf + I + G) for the partial 18S rRNA gene were run with four chains for 4 x 10^6^ generations. A combined analysis of the three ribosomal genes was not undertaken due to some sequences not being available for all species. The sampling for Markov chains was carried out at intervals of 100 generations. For each analysis, two runs were conducted. After discarding burn-in samples of 30% and evaluating convergence, the remaining samples were retained for more in-depth analyses. The topologies were used to generate a 50% majority-rule consensus tree. On each appropriate clade, posterior probabilities (PP) were given. FigTree software version v.1.4.3 [86] was used for visualizing trees from all analyses.

Haplotype network for D2-D3 expansion segments of 28S rRNA and COI gen was conducted on *C. annuliferum* species complex using Population Analysis with Reticulate sequences from Trees (PopART) software available at http://popart.otago.ac.nz (accessed on 27 December 2022) using Templeton, Crandall and Sing (TCS) option [87,88].

## 5. Conclusions

This research verified the importance of using integrative taxonomy for ring nematode species, and, in particular, on the identification of *Criconema* species. The blind-identification test, as well as morphometrics and molecular analyses, confirmed the presence of hyper-cryptic biodiversity within the *C. annuliferum* complex, broadening and increasing the diversity of this group of nematodes in Spain. In summary, our results demonstrate, for the first time, the existence of a hyper-cryptic species complex within *Criconema* comprising four well separated species, *viz*. *C. annuliferum*, *C. paraannuliferum*, *C. plesioannuliferum* and *C. pseudoannuliferum* sp. nov. Then, our data support our hypothesis that we have only deciphered just a negligible part of the species diversity within criconematids reported in southern Spain in natural habitats and possibly worldwide. Additional sampling efforts are needed to give the significant gaps in soil plant-parasitic nematode biodiversity regarding the potential number of undescribed species and the hypothesis suggesting the Iberian Peninsula as a possible centre of speciation for some groups, including Longidoridae, Paratylenchidae and Criconematidae [13,33,54,63,89].

## Figures and Tables

**Table 1:** Eigenvector and SS loadings of factor analysis from morphometric characters for *Criconema annuliferum* species complex (*C. annuliferum*, *C. paraannuliferum*, *C. plesioannuliferum*, *C. pseudoannuliferum* sp. nov.) ^a^.

Character/Ratio ^b^	MLC1	MLC2	MLC3
L	0.86	0.25	0.30
Stylet length	0.66	0.00	0.36
R	0.63	0.25	0.57
RV	−0.06	0.85	0.20
Ran	−0.22	0.90	−0.19
a	0.67	0.03	−0.04
b	0.61	−0.33	0.16
c′	0.13	0.81	−0.57
V	0.24	−0.13	0.64
SS loadings	2.52	2.43	1.38
% of total variance	0.28	0.27	0.15
Cumulative % of total variance	0.28	0.55	0.70

^a^ Based-on populations reported by Clavero-Camacho et al. [13] and 21 female specimens of *Criconema pseudoannuliferum* sp. nov. from paratype population sample of maritime pine. Values of morphometric variables 1 to 3 (eigenvalue > 0.64) are underlined. All populations were molecularly identified. ^b^ Morphological and diagnostic characters according to Hunt & Palomares-Rius [35].

**Table 2:** Parameters evaluating *Criconema annuliferum species complex* delimitation based on two rRNA genes (D2-D3 expansion segments of the 28S rRNA, ITS) and one mtDNA barcoding locus, COI for four *Criconema* species of the complex.

Species	Gene	Intra/Inter ^a^	P ID (Liberal) ^b^	Clade Support ^c^	Rosenberg’s P_AB_ ^d^
*Criconema annuliferum*	D2-D3	0.19	**0.95 (0.85, 1.0)** ^e^	**1.00**	**5.1 × 10^−5^**
	ITS	-	**-**	**-**	**-**
	COI	0.02	**1.00 (0.95, 1.0)**	**1.00**	**2.1 × 10^−5^**
*Criconema paraannuliferum*	D2-D3	0.14	**0.98 (0.95, 1.0)**	**1.00**	**7.1 × 10^−9^**
	ITS	0.05	**0.99 (0.97, 1.0)**	**1.00**	**2.5 × 10^−11^**
	COI	0.05	**1.00 (0.97, 1.0)**	**1.00**	**6.5 × 10^−20^**
*Criconema plesioannuliferum*	D2-D3	0.09	**0.98 (0.93, 1.0)**	**0.98**	**7.1 × 10^−9^**
	ITS	0.02	**1.00 (0.96, 1.0)**	**1.00**	**2.5 × 10^−11^**
	COI	0.07	**1.00 (0.98, 1.0)**	**1.00**	**2.6 × 10^−5^**
*Criconema pseudoannuliferum* sp. nov.	D2-D3	0.05	**0.99 (0.93, 1.0)**	**1.00**	**5.1 × 10^−5^**
	ITS	0.01	**0.98 (0.88, 1.0)**	**1.00**	**1.1 × 10^−8^**
	COI	0.02	**1.00 (0.95, 1.0)**	**1.00**	**3.6 × 10^−6^**

^a^ Intra-species variation relative to inter-species variation. ^b^ The P ID (Liberal) value represents the probability (with the 95% confidence interval) for the prediction, of making a correct identification of an unknown specimen of the focal species using DNA Barcoding (closest genetic distance). P ID (Liberal) values ≥ 0.93 considered to be delimited [37]. Numbers in bold represent significant values. ^c^ Clade support: posterior probabilities from Bayesian trees. ^d^ Rosenberg’s P_AB_ value is the probability that the monophyly of a group of sequences is the result of random branching. ^e^ Significant results are indicated in bold. (-) Not obtained or not performed because of the lack of ITS for this species in NCBI.

**Table 3:** Morphometrics of *Criconema pseudoannuliferum* sp. nov. from the rhizosphere of maritime pine (*Pinus pinaster* Ait.) in Casares, Málaga province, Spain.

Character/Ratio ^a^	Holotype	Female Paratypes	Males	Fourth-Stage Juveniles
n	1	20	2	4
L	641	633.6 ± 54.1 (526–715)	(486, 597)	503.4 ± 14.4 (486–520)
R	70	71.5 ± 2.8 (67–79)	-	73.3 ± 2.8 (70–76)
Rst	16	14.0 ± 1.5 (12–16)	-	17.0 ± 1.8 (15–19)
Roes	21	19.3 ± 1.6 (17–22)	-	21.5 ± 1.3 (20–23)
Rex	22	21.0 ± 1.4 (19–23)	-	23.0 ± 1.2 (22–24)
RV	9	9.6 ± 0.7 (9–11)	-	-
Rvan	5	4.7 ± 0.6 (4–6)	-	-
Ran	4	4.8 ± 0.5 (4–6)	-	4.5 ± 0.6 (4–5)
O	9.1	9.3 ± 0.9 (7.5–11.0)	-	10.0 ± 0.6 (9.2–10.5)
a	9.3	11.4 ± 1.8 (8.6–14.2)	(16,8, 23.9)	9.8 ± 1.5 (8.5–11.8)
b	3.9	3.9 ± 0.3 (3.4–4.4)	(3.8, 4.9)	3.5 ± 0.1 (3.4–3.6)
c	25.6	24.7 ± 3.2 (19.6–29.5)	(14.6, 16.2)	18.4 ± 1.0 (17.0–19.4)
c′	1.0	1.2 ± 0.1 (1.0–1.4)	(1.4, 2.1)	1.2 ± 0.1 (1.0–1.2)
V or T	87.7	89.3 ± 1.1 (87.7–91.7)	(28.6, 37.0)	-
VL/VB	1.4	1.6 ± 0.2 (1.3–1.9)	-	-
First annulus	23.0	21.8 ± 1.1 (20.0–23.5)	-	19.3 ± 1.0 (18.0–20.0)
Second annulus	21.0	20.0 ± 1.0 (18.0–21.5)	-	17.0 ± 0.8 (16.0–18.0)
Stylet	110.0	109.7 ± 3.0 (105.0–116.0)	-	96.0 ± 2.9 (93.0–99.0)
Conus	90.0	91.0 ± 2.3 (87.0–97.0)	-	80.3 ± 1.7 (78.0–82.0)
Pharynx	165.0	164.2 ± 7.3 (152–177)	(121, 127)	145.0 ± 3.7 (140–149)
Max. body width	69.0	56.5 ± 7.0 (43.0–69.0)	(25.0, 29.0)	52.0 ± 14.4 (43.0–59.0)
Anal body diam.	24.0	22.6 ± 2.6 (18.0–27.0)	(20.0, 21.5)	23.6 ± 2.3 21.0–26.0)
Vulva to anus distance	43.0	42.2 ± 4.6 (34.0–50.0)	-	-
Tail	25.0	26.0 ± 3.1 (20.0–32.0)	(30.0, 41.0)	27.5 ± 2.1 (25.0–30.0)
Spicules	-	-	(47.0, 48.0)	-
Gubernaculum	-	-	(9.0, 10.0)	-

^a^ Measurements are in µm and in the form: (mean) ± (standard deviation), (range). (-) Not obtained or not performed. Abbreviations: a, body length/maximal body width; b, body length/pharyngeal length; c, body length/tail length; c′, tail length/body width at anus; L, (total body length); n, number of specimens studied; O, distance between stylet base and orifice of dorsal oesophageal gland as percentage of stylet length; R, total number of body annuli; Roes, number of annuli in pharyngeal region; Rex, number of annuli between anterior end of body and excretory pore; Rst, number of body annuli between labial disc and stylet knobs; RV, number of annuli between posterior end of body and vulva; Rvan, number of annuli between vulva and anus; Ran, number of annuli on tail; V, (distance from anterior end to vulva/body length) × 100; VL/VB, distance between vulva and posterior end of body divided by body width at vulva; T, (distance from cloacal aperture to anterior end of testis/body length) × 100.

## Data Availability

The datasets generated during and/or analysed during the current study are available NCBI and from the corresponding author on reasonable request.
